# Impacts of Cu-Doping on the Performance of La-Based RRAM Devices

**DOI:** 10.1186/s11671-019-3064-1

**Published:** 2019-07-09

**Authors:** Yongte Wang, Hongxia Liu, Xing Wang, Lu Zhao

**Affiliations:** Key Laboratory for Wide Band Gap Semiconductor Materials and Devices of Education, School of Microelectronics, Xidian University, Xi’an, 710071 China

**Keywords:** LaAlO_3_, RRAM, Cu-doping, Annealing, Resistive switching characteristics

## Abstract

In this paper, the effects of Cu insertion layer and rapid thermal annealing on the resistive switching behaviors of La-based resistive switching access memory (RRAM) devices have been investigated. Compared with the undoped control sample (Cu/LaAlO_3_/Pt), the Cu-embedded devices show higher device yield and reset stop voltage, which indicates that the reliability of La-based RRAM has been effectively improved. However, the unannealed Cu/LaAlO_3_: Cu/Pt RRAM device still suffers from serious dispersion of parameters. It was proved that the RRAM device with Cu insertion layer and annealing treatment exhibits the best resistive switching characteristics such as low forming voltage, high on/off ratio and fine electrical uniformity. These improvements can be attributed to the diffusion of Cu atoms and the formation of Cu nanocrystals (Cu-NCs) after annealing process, since the diffused Cu atoms and the Cu-NCs could enhance the local electric field and weaken the randomness of the formation/rupture of conductive filaments.

## Introduction

Resistive random access memory (RRAM) is considered as a development direction for the next-generation non-volatile memory devices, which has been attracting much attention due to its simple structure, low power consumption, high scalability, fast operation speed and multi-value storage capacity [1]. RRAM is often fabricated into a metal–insulator–metal (M–I–M) sandwich structure, and the intermediate dielectric layer has a significant influence on its resistive switching (RS) performance. Thus, a wide variety of materials, including many common used high dielectric constant (high-*k*) materials (such as HfO_2_ [2], Al_2_O_3_ [3], and ZrO_2_ [4]), have been investigated extensively for RRAM applications. Among all the oxide materials, lanthanum-based oxide is one of the most promising high-*k* dielectric materials, which has raised great research interests due to its high-*k* value, large band gap, and fine thermal stability [5]. Recently, good resistance switching characteristics, such as low operating voltage, high resistance window, long holding time, long cycle endurance, and good consistency, have been found in La-based RRAMs, indicating the potential application of La-based high-*k* materials in RRAMs [6, 7].

Also, owing to the advantages of excellent uniformity, precise thickness control and compatibility with CMOS process, atomic layer deposition (ALD) technology has been one of the most commonly used growth methods to produce La-based dielectric films [8]. Unfortunately, undesirable high forming voltages are always required in ALD-deposited RRAM devices because of the good quality dielectric films, which may lead to a high failure rate, low on/off ratio, poor endurance, and wide dispersion of the devices [9]. In order to obtain RRAM devices with better RS performance, materials/device structure engineering, including ion implantation [10], dopant diffusion [11], or inserting nanocrystals (NCs) [12], need to be adopted in the ALD-prepared La-based RRAMs.

In recent reports, different doping technologies for improving the RS behaviors of the traditional high-*k* materials (HfO_2_ [13], ZrO_2_ [14], etc.) have been extensively studied. However, the NC-improved RS behaviors of La-based RRAM devices have not been reported so far. Thus, a Cu-embedded LaAlO_3_ device with the structure of Cu/LaAlO_3_/Cu/LaAlO_3_/Pt is fabricated for the memory application, and attention was focused on the impacts of Cu-doping on the performance and switching mechanism of La-based RRAM devices.

## Methods

**Fig. 1:**
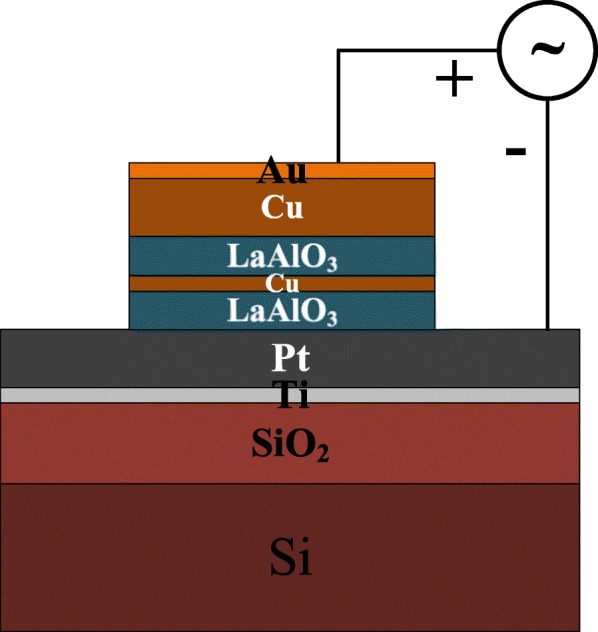
Schematic diagram of Cu-embedded LaAlO_3_ device with the structure of Cu/LaAlO_3_/Cu/LaAlO_3_/Pt

The schematic diagram of the fabricated device with the structure of Cu/LaAlO_3_/Cu/LaAlO_3_/Pt is shown in Fig. 1. The fabrication process of La-based RRAM device is as follows: A bilayer metal, 100-nm Pt/10-nm Ti, was first deposited on a 2-in. SiO_2_/Si wafer as the bottom electrode (BE) by electron beam evaporation. Subsequently, the temperature of Picosun R-150 ALD reactor was set as 300 °C, and ~ 10-nm LaAlO_3_ (La/Al ratio as 3:1) thin film was deposited on the Pt/Ti/SiO_2_/Si substrates, using La(^i-^PrCp)_3_ as La precursor, Al(CH_3_)_3_ as Al precursor and O_3_ as the oxidant. Then, ~ 2-nm Cu layer was grown on the LaAlO_3_ at a rate of 0.1 Å/s using an electron beam evaporator (EBE). Again, ~ 10 nm LaAlO_3_ (La/Al ratio as 3:1) thin film was deposited by ALD at 300 °C. After the LaAlO_3_/Cu/LaAlO_3_ switching layer had been prepared by using the ALD-EBE-ALD process, rapid thermal annealing (RTA) process was carried out in an N_2_ ambient at 600 °C for 30 s. The top electrode (TE) of 10 nm Au/150 nm Cu was deposited on the LaAlO_3_ dielectric by electron beam evaporation after lithography, and followed by peeling off to fabricate devices which have sizes from 50 × 50 μm^2^ to 250 × 250 μm^2^. In order to further understand the impacts of Cu-doping on the performance of La-based RRAM devices, two control samples, S1: Au/Cu/LaAlO_3_/Pt (unannealed) and S2: Au/Cu/LaAlO_3_: Cu/Pt (unannealed), were set up. And the sample with Au/Cu/LaAlO_3_: Cu-NC/Pt structure was assigned as S3.

X-ray photoelectron spectroscopy (XPS) was used to analyze the distribution of the doped Cu atoms and cross-sectional transmission electron microscopy (TEM) was used to observe the microstructure of the fabricated RRAM devices. The RS properties were measured under different modes using an Agilent B1500A semiconductor parameter analyzer. A current compliance of 1 mA was imposed to protect the fabricated device units from damages of high currents during forming and set processes.

## Results and Discussion

**Fig. 2:**
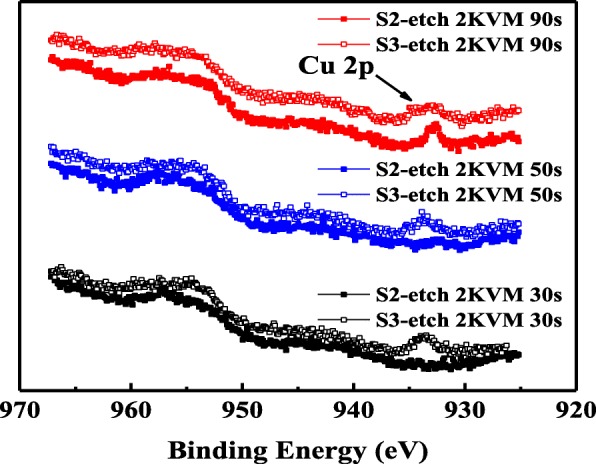
XPS results of Cu 2p spectra for S2 and S3 after Ar ion etching for 30 s, 60 s, and 90 s

Figure 2 shows the X-ray photoelectron spectroscopy (XPS) depth analysis of Cu 2p spectra in the Cu-doped LaAlO_3_ film (Etching parameters: 2 KVM Ar ion, ~ 1 Å/s etching rate). As can be seen in Fig. 2, Cu 2p peak can hardly be found in the unannealed sample (S2) after etching for 30 s or 60 s, while after etching for 90 s, a notable Cu 2p peak appears, indicating that the Cu atoms mainly concentrate in the Cu-embedded layer. Differently, the Cu atoms are observed in the whole LaAlO_3_ film after annealing treatment, i.e. after etching for 30 s, 60 s, and 90 s, obvious Cu 2p peaks can be observed in S3. The XPS results confirm that high-temperature annealing will lead to redistribution of the doped Cu atoms, which may help to improve the electrical characteristics of La-based RRAMs.

**Fig. 3:**
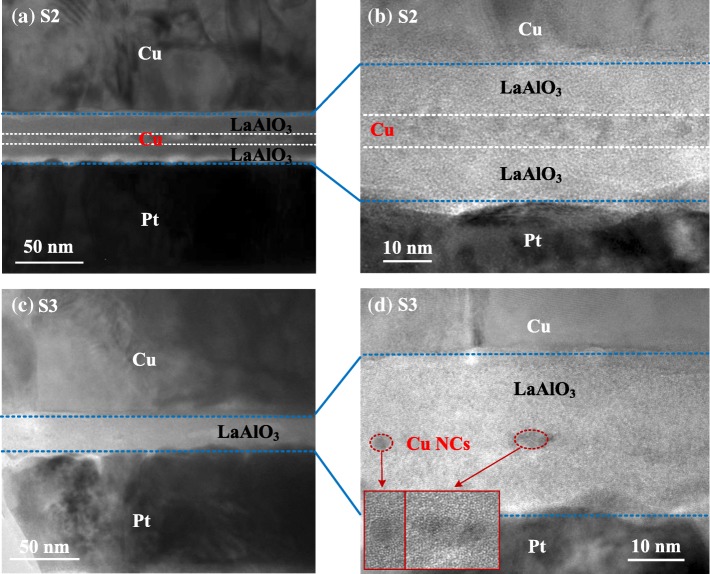
TEM images of the Cu-embedded RRAM devices. **a** A typical cross-sectional TEM image of S2. **b** A HRTEM image of S2. **c** A cross-sectional TEM image of S3. **d** A HRTEM image of S3

Figure 3 shows the typical cross-sectional transmission electron microscope (TEM) image of the two Cu-embedded LaAlO_3_ RRAMs (i.e. S2 and S3). As shown in Fig. 3a, the laminated structure of the unannealed Cu/LaAlO_3_/Cu/LaAlO_3_/Pt device could be recognized clearly in the TEM image of S2. It is worth noting that after the deposition of the upper LaAlO_3_ layer at 300 °C of the ALD process, the embedded ~ 2-nm Cu nanolayer has been slightly affected by thermal diffusion. Therefore, from the high-resolution image of Fig. 3b, the irregular and separated Cu nanoparticles with the size of 2~6 nm embedded in LaAlO_3_ layer can be clearly observed. The additional annealing treatment after ALD process would further enhance the thermal diffusion of Cu atoms, making it difficult to distinguish the existence of Cu nanolayer as shown in Fig. 3c. With the help of higher resolution TEM image as shown in Fig. 3d, an approximate of 25-nm-thick LaAlO_3_ layer embedded with several spherical and separated Cu-NCs could be observed, indicating that part of the Cu nanolayer has already diffused into the LaAlO_3_ dielectric with some smaller sized Cu-NCs being left behind after 600 °C annealing treatment.

**Fig. 4:**
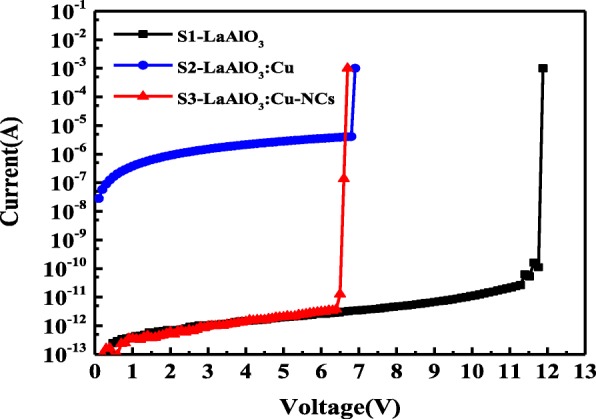
Forming process of the three kinds of La-based RRAM

The electrical forming process of samples S1, S2, and S3 are shown in Fig. 4. As can be seen in Fig. 4, a high voltage of about 12 V is required in the forming process of S1, and a much lower forming voltage (~ 7 V) is needed in S2 and S3, showing that the forming voltage of the La-based devices can be effectively reduced by inserting a Cu nanolayer in the dielectric film. In addition, compared with the initial resistance value of S1 (2.51 × 10^12^ Ω, read at 1 V), the resistance of S2 is much lower (2.65 × 10^6^ Ω, read at 1 V), and this value increases after annealing process (S3, 2.83 × 10^12^ Ω, read at 1 V). The above variations of the forming voltage and initial resistance can be attributed to the changes of dielectric properties of LaAlO_3_ films through materials/device structure engineering. Due to the excellent quality of La-based dielectric films prepared by ALD method, an extremely high electric field strength is needed to break the insulator down (i.e., S1). After the Cu nanolayer was inserted in the dielectric film, the ALD-grown high-quality switching layer will be affected by this metal nanolayer, which would make the dielectric easier to break down, and ultimately leads to a much lower forming voltage in S2. Also, the energy barrier of the oxygen vacancy formation could be effectively decreased and more meta-stable defects would be brought into the dielectric film because of the structural differences between Cu and LaAlO_3_ materials (lattice match, thermal expansion match, etc.) [15]. Consequently, a larger number of defects (charge traps, metal ions, oxygen vacancies, etc.) would be introduced into the LaAlO_3_ resistive switching layer, leading to the reduction of the initial resistance of S2 [16]. However, these large numbers of defects in the dielectric thin films of S2 could be effectively reduced (or eliminated) by additional annealing treatment, leading to a high initial resistance of S3 [17]. In addition, the additional annealing treatment has brought some Cu-NCs and diffused Cu atoms into the LaAlO_3_ dielectric films, which would further enhance the local electric field and result in a low forming voltage of S3 [18].

**Fig. 5:**
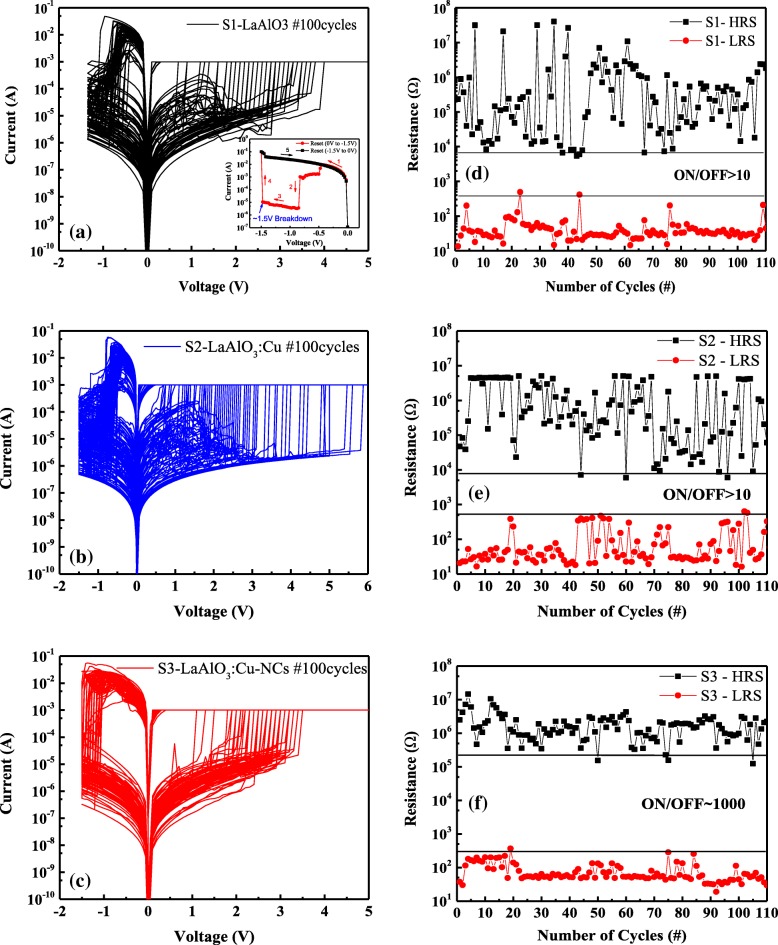
**a**–**c** Typical bipolar *I*–*V* curves and **d**–**f** the endurance test of S1, S2, and S3

Typical bipolar current–voltage (*I*–*V*) curves of ~ 100 direct current (DC) sweep cycles for the three kinds of La-based RRAM devices (area size of 50 μm × 50 μm) are obtained in Fig. 5a–c. The test voltage (0 to − 1.5 V and − 1.5 to 0 V for the reset process; 0 to 5 V and 5 to 0 V for the set process) is biased on the TE (Cu) while the BE (Pt) is grounded. Although three La-based RRAM devices display 100 consecutive cycles of repeatable bipolar RS behavior, there are some great differences among them. Firstly, compared with S2 and S3, the undoped sample S1 is easier to be damaged during the cyclic set–reset operations with the reset stop voltage (*V*_stop_) of − 1.5 V (as shown in the insertion of Fig. 5a), which indicates that the maximum *V*_stop_ of S1 is lower than those of S2 and S3. Considering this, a relatively low *V*_stop_ of − 1.4 V is used in the case of sample S1. Another difference is that the *I*–*V* curves of S1 and S2 show abnormal fluctuations (rise and fall) during the set process, which is quite different from the smooth *I*–*V* curves of S3. This phenomenon is closely related to the remnant of some conductive filaments (CFs) in the undoped (or doped but unannealed) LaAlO_3_ dielectric films after the reset process. Besides, compared with S1 and S2, S3 has *I*–*V* curves showing more consistency and smaller distribution of set/reset voltages, implying that the stability of the RRAM devices can be effectively improved by doping and annealing treatment. Figure 5d–f are the endurance test (~ 100 cycles, read at − 0.1 V) of S1–S3 extracted from the left of Fig. 5a–c. The maximum resistance ratio of Cu/LaAlO_3_/Pt RRAM devices, namely, the maximum high resistance state (HRS) to the minimum low resistance state (LRS) can be as high as 6 orders of magnitude. However, the wide random fluctuation in HRS of S1 and S2 brings about a very low level on/off window (~ 10). Unlike S1 and S2, the on/off window of S3 is about 100 times larger than that of S1 and S2, indicating that the consistency characteristic of Cu-doped La-based RRAM devices is effectively improved after annealing. The annealing treatment in S3 not only causes the Cu atoms to diffuse around the entire LaAlO_3_ film but also forms Cu-NCs in the dielectric. Thus, the local electric field has been enhanced; the randomness of the CF formation/rupture has been controlled, and the HRS (LRS) distribution has been improved [19]. The above results suggest that the idea of embedding a Cu nanolayer into the La-based RRAM requires a certain degree of thermal treatment in order to achieve better device performance.

**Fig. 6:**
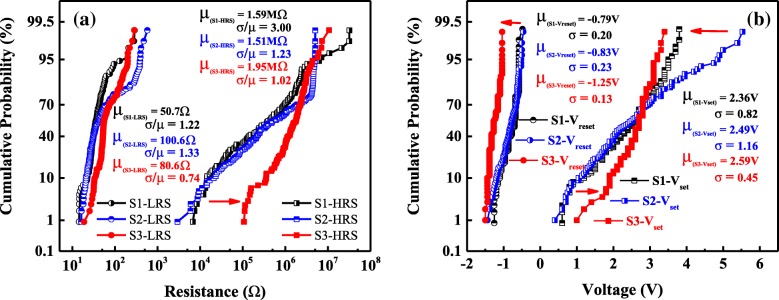
Cumulative probability of **a** HRS and LRS (read at − 0.1 V) and **b** set and reset voltages

Figure 6 a shows the cumulative probability of the HRS and LRS resistances (read at − 0.1 V), and Fig. 6b records the cumulative probability of the set and reset voltages. In Fig. 6a, the mean values (*μ*) of LRS and HRS in S1, S2, and S3 are obtained to be 50.7 Ω and 1.59 MΩ, 100.6 Ω and 1.51 MΩ, and 80.6 Ω and 1.95 MΩ, respectively. However, the coefficient of variation (*σ*/*μ*) of LRS and HRS vary greatly when compared with the roughly similar mean values in S1, S2, and S3. Among them, S3 has the minimum σ/μ value (LRS − 0.74, HRS − 1.02), followed by S2 (LRS − 1.33, HRS − 1.23), and the σ/μ of S1 is the worst (LRS − 1.22, HRS − 3.00). As shown in Fig. 6b, the mean values of the reset/set voltages are about − 0.79 V/2.36 V, − 0.83 V/2.49 V, and − 1.25 V/2.59 V for the samples S1, S2, and S3 respectively. The standard deviation (*σ*) of the reset/set voltages, which is used to evaluate the dispersion of parameters, are found to be 0.20/0.82 (S1), 0.23/1.16 (S2), and 0.13/0.45 (S3), respectively. It can be found that wide variations of HRS, LRS, *V*_set_, and *V*_reset_ in S1 and S2 are improved after annealing. Compared with S1 and S2, the doped and annealed one (S3) exhibits better uniformity, indicating that S3 has the best operation stability among the three. As mentioned above, large numbers of defects are likely to be introduced into S2, which will cause problems with the reliability and stability of the devices. For S3, those large numbers of defects are eliminated by the thermal process, and the formation/rupture randomness of the CFs is reduced due to the existence of Cu-NCs. Thus, fine uniformity with small variations in switching voltages and resistance values are obtained in S3.

**Fig. 7:**
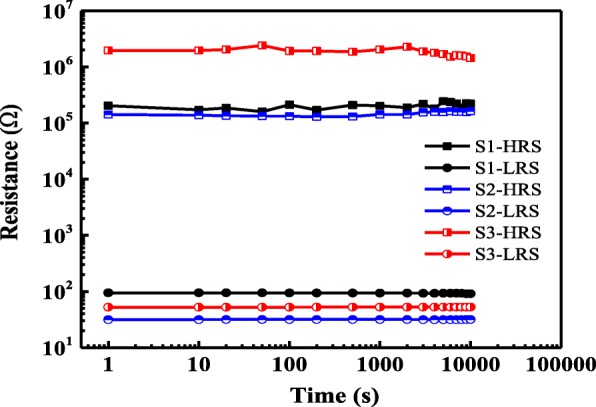
Retention behaviors of La-based RRAM devices at room temperature

**Fig. 8:**
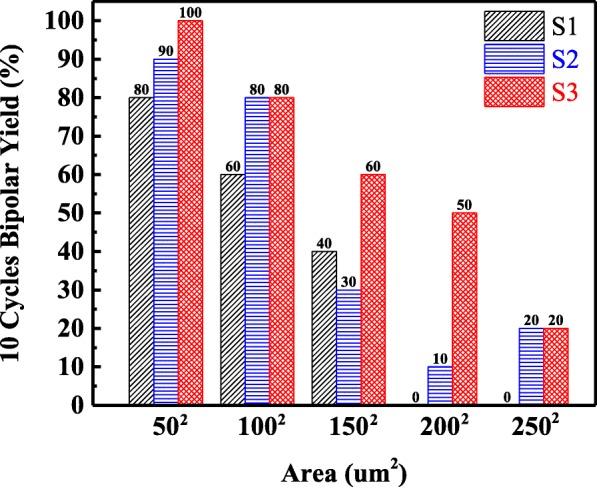
DC SET/RESET 10-cycle bipolar yield of La-based RRAM devices

The retention characteristics of the three kinds of La-based RRAM devices under read-out voltage of − 0.1 V at room temperature are illustrated in Fig. 7. During the retention test, the Cu/LaAlO_3_: Cu-NC/Pt devices exhibit stable retention performance for over 10^4^ s at room temperature with a nearly constant *R*_HRS_/*R*_LRS_ ratio of up to three orders of magnitude, conforming the nonvolatile characteristics of the La-based RRAMs. DC SET/RESET 10-cycle bipolar yield is measured for evaluating the switching ability of S1, S2, and S3. As shown in Fig. 8, S3 has the best yield, followed by S2, and S1 is the worst. This result shows that an embedded Cu layer is helpful to increase the yield of La-based RRAMs, and the yield of the devices can be further improved by additional thermal treatment. Moreover, it can be found in Fig. 8 that the yields of the devices increase with the decreased device area. This phenomenon indicates that the resistive switching mechanism of Cu/LaAlO_3_/Pt RRAM devices may be closely related to the Joule heat effect, that is, Joule heat participates in the formation/rupture of conductive filaments and seems to be more prominent in smaller size devices.

**Fig. 9:**
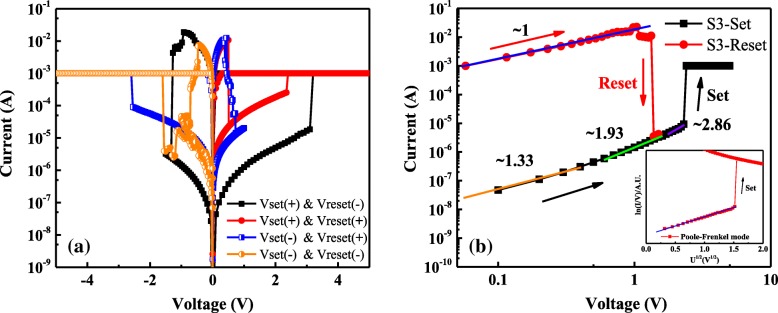
**a** *I*–*V* measurement of S3 in different voltage directions. **b** Double logarithmic plotting of *I*–*V* curves and linear fittings of S3, and the insert shows the ln(*I*/*V*)–*V*^1/2^ plotting of the set process

In order to gain more insights into the Cu-NC–treated La-based RRAMs, further analysis has been focused on the resistance switching mechanism of S3. As shown in Fig. 9a, setting and resetting voltages in different directions are applied in the *I*–*V* measurement of S3. The test results show that S3 has both unipolar and bipolar resistive switching behaviors, indicating the Cu-NC–treated La-based RRAMs are nonpolar. Researchers believe the nonpolar (unipolar) resistive switching behaviors are closely related to the Joule heat-assisted formation/rupture of CFs [7]. In the reset process of La-based RRAM, a high current overshoot phenomenon is observed and then the Joule heating effect is induced, leading to melting, sintering, or thermal oxidation of CFs. Figure 9b shows the double-logarithmic plotting of *I*–*V* curves and linear fittings of S3, and the insert shows the ln(*I*/*V*)–*V*^1/2^ curve of the set process. Obviously, the *I*–*V* relationship in LRS exhibits an Ohmic conduction behavior with a slope of about 1, implying the existence of CFs in the dielectric after the set process. However, the conducting mechanism of HRS is slightly complicated, and the *I*–*V* curves at HRS can be divided into three straight lines with three different slopes. In the low voltage region (< 0.8 V, orange line), the slope of the fitting line is about 1.33, which is close to the Ohmic transport mechanism. With the increase of voltage (~ 0.8 to ~ 2 V, green line), the slope of the fitting line increases to 1.93 (*I*~*V*^1.93^), which conforms to Child’s square law (*I*~*V*^2^). In the third region (> 2 V, purple line), the slope of fitting line will continue to increase (e.g., 2.86 in this case), and the current will increase sharply when *V*_set_ is reached. The conduction mode of HRS, which is composed of Ohmic transport region and Child’s law region, is in good agreement with the classical space charge limited current (SCLC) mechanism [20, 21]. The appearance of SCLC conduction mechanism indicates the formation and rupture of local conduction path [22], which is regarded as the main RS mechanism of Cu/LaAlO_3_: Cu-NC/Pt devices. Besides, the HRS conduction is also found to fit well with the Poole–Frenkel conduction mechanism (the insert). The Poole–Frenkel effect is mainly caused by the electric field excited carriers hopping through the trapped states [23], which suggests that there is still a large number of defects in the LaAlO_3_ films even after annealing treatment.

**Fig. 10:**
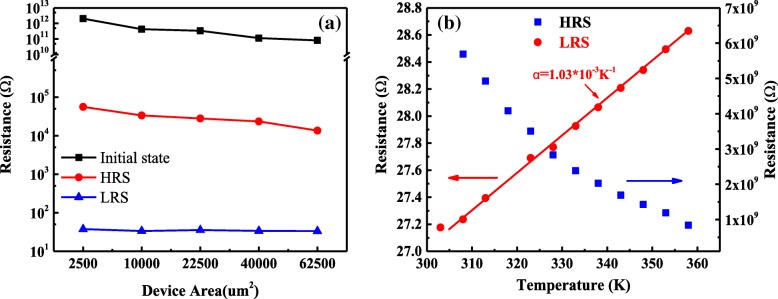
**a** Electrode area dependence of the HRS and LRS. **b** Temperature dependence of the HRS and LRS

Dependence of S3 on electrode area (Fig. 10a) and temperature (Fig. 10b) has been studied to further understand the RS mechanism. It can be seen from Fig. 10a that the LRS resistance is independent of the electrode area, while the HRS resistance (and initial resistance) decreases with the increase of device area, which indicates that the RS mechanism of S3 originates from the formation and rupture of CFs. In Fig. 10b, the HRS resistances decrease with increasing temperature, showing that the OFF-state of S3 can be associated with a semiconducting behavior. In contrast, the LRS resistances increase with increasing temperature, indicating a metallic characteristic in ON state [24]. According to the literature, the relationship between the metal resistance and the temperature is usually studied by the equation of *R*(*T*) = *R*_0_[1 + *α*(*T* − *T*_0_)] [25]. And the blue linear fit in Fig. 10b determines the temperature coefficient (*α*) to be 1.03 × 10^−3^ K^−1^. This value is slightly smaller than the reported values of the Cu nanowires in other literatures (2.5 × 10^−3^ K^−1^ [26], 2.39 × 10^−3^ K^−1^ [27]). Owing to the fact that large numbers of defects have been introduced into the Cu-NC–doped LaAlO_3_ films, a lower *α* value of Cu CFs is obtained in this paper.

**Fig. 11:**
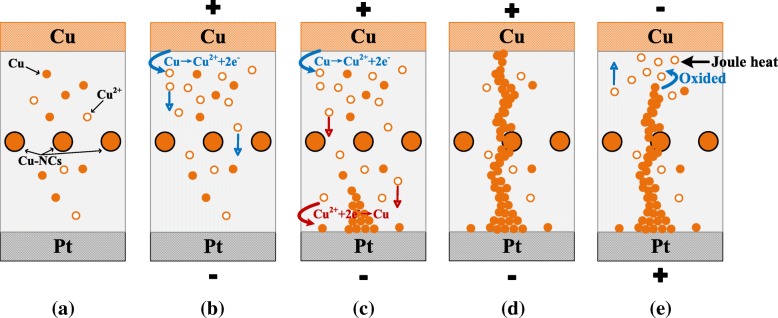
Schematic diagrams for the RS mechanism of Cu/LaAlO3: Cu-NC/Pt devices in **a** initial state; **b**, **c** set process; **d** ON state; and **e** reset process

Therefore, a reasonable explanation has been proposed for the RS effect of Cu/LaAlO_3_: Cu-NC/Pt devices in our case. The formation and rupture of Cu CFs is very likely to be mediated by electrochemical metallization (ECM) and Joule heat effect. Figure 11 shows schematic diagrams for the RS mechanism of Cu/LaAlO3: Cu-NC/Pt devices in (a) initial state; (b), (c) Set process; (d) ON state; and (e) Reset process. When a positive voltage is applied to TE (Cu), an oxidation reaction, which is described as Cu → Cu^2+^ + 2e^−^, occurs on the electrochemically active material (Fig. 11b). Under the action of the electric field, the mobile Cu^2+^ cations migrate toward BE (Pt) through LaAlO_3_ film, and a reduction reaction of Cu^2+^ + 2e^−^ → Cu occurs at the cathode (Fig. 11c). It is worth noting that there are some Cu-NCs and diffused Cu atoms in the La-based dielectrics of S3, which is the natural pathways for the formation of Cu CFs. Thus, the continuously precipitated Cu metal atoms will tend to grow along these natural pathways and eventually reach the TE to form a conductive channel (Fig. 11d). When the polarity of applied voltage is reversed, the dissolution process, which closely relates to electrochemical effect and Joule heat effect, occurs somewhere along the filament, resulting in an almost complete fracture of the CFs and the device into the OFF state (Fig. 11e).

## Conclusion

In summary, a metal-doped method is introduced to improve the performance of La-based RRAM devices. Obvious improvements of the resistive switching characteristics, including lower forming voltage, higher on/off ratio, better electrical uniformity, and superior device yield, are corroborated by the *I*–*V* measurement results of the Cu-doped and annealed sample. XPS and TEM analysis results confirmed that the improvements of switching performance could be attributed to the diffusion of Cu atoms and the formation of Cu nanocrystals (Cu-NCs) after the annealing process. Further studies reveal that the resistive switching mechanism of Cu\LaAlO_3_: Cu-NC\Pt devices can be attributed to the formation and rupture of Cu conductive filaments, which is closely related to the SCLC mechanism and Joule heating effect. This study demonstrates a feasible method to control the resistive switching behaviors of the RRAMs by embedding Cu nanocrystals, and more works need to be done for understanding the physical mechanism and the inherent laws of La-based RRAMs.

## Data Availability

The datasets supporting the conclusions of this manuscript are included within the manuscript.

## Abbreviations

ALD: atomic layer deposition; RRAM resistive random access memory; NCs nanocrystals; M-I-M metal-insulator-metal; RS resistive switching; High-*k* high dielectric constant; RS resistive switching; BE bottom electrode; RTA rapid thermal annealing; TE top electrode; XPS X-ray photoelectron spectroscopy; DC direct current; CFs conductive filaments; HRS high resistance state; LRS low resistance state; SCLC space charge limited current
